# 4-Sulfamoylanilinium chloride

**DOI:** 10.1107/S1600536810019471

**Published:** 2010-05-29

**Authors:** Donia Zaouali Zgolli, Habib Boughzala, Ahmed Driss

**Affiliations:** a Laboratoire de Matériaux et Cristallochimie, Faculté des Sciences, El Manar, 2092 Tunis, Tunisia

## Abstract

In the crystal structure of the title compound, C_6_H_9_N_2_O_2_S^+^·Cl^−^, the chloride anions are sandwiched between layers of 4-sulfonamido­anilinium anions. The components interact by way of N—H⋯Cl and N—H⋯O hydrogen bonds, building up a three-dimensional network.

## Related literature

For the biological activity of diamines, see: Pasini & Zunino (1987 ▶); Otsuka *et al.* (1990 ▶); Michalson & Smuszkovicz (1989 ▶); Reedijk *et al.* (1996 ▶). For their use in asymmetric catalysis, see: Blaser (1992 ▶). For related structures, see: Chatterjee *et al.* (1981 ▶); Gelbrich *et al.* (2008 ▶); Gelmboldt *et al.* (2004 ▶); Smith *et al.* (2001 ▶). 
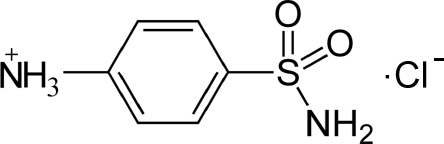


## Experimental

### 

#### Crystal data

C_6_H_9_N_2_O_2_S^+^·Cl^−^
*M* *_r_* = 208.66
Orthorhombic, 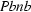

*a* = 7.4608 (2) Å
*b* = 7.7278 (2) Å
*c* = 31.694 (2) Å
*V* = 1827.35 (13) Å^3^
*Z* = 8
Mo *K*α radiation
μ = 0.61 mm^−1^
*T* = 298 K
0.30 × 0.20 × 0.10 mm

#### Data collection

Enraf–Nonius CAD-4 diffractometer
Absorption correction: ψ scan (North *et al.*, 1968 ▶) *T*_min_ = 0.982, *T*_max_ = 0.994
2906 measured reflections
1989 independent reflections
1442 reflections with *I* > 2σ(*I*)
*R*_int_ = 0.033
2 standard reflections every 120 min  intensity decay: none

#### Refinement

*R*[*F* ^2^ > 2σ(*F* ^2^)] = 0.042
*wR*(*F* ^2^) = 0.182
*S* = 1.07
1989 reflections
110 parameters
H-atom parameters constrained
Δρ_max_ = 0.42 e Å^−3^
Δρ_min_ = −0.44 e Å^−3^

### 

Data collection: *CAD-4 EXPRESS* (Enraf–Nonius, 1994 ▶); cell refinement: *CAD-4 EXPRESS*; data reduction: *XCAD4* (Harms & Wocadlo, 1995 ▶); program(s) used to solve structure: *SHELXS97* (Sheldrick, 2008 ▶); program(s) used to refine structure: *SHELXL97* (Sheldrick, 2008 ▶); molecular graphics: *ORTEP-3 for Windows* (Farrugia, 1997 ▶); software used to prepare material for publication: *WinGX* (Farrugia, 1999 ▶).

## Supplementary Material

Crystal structure: contains datablocks I, global. DOI: 10.1107/S1600536810019471/dn2568sup1.cif

Structure factors: contains datablocks I. DOI: 10.1107/S1600536810019471/dn2568Isup2.hkl

Additional supplementary materials:  crystallographic information; 3D view; checkCIF report

## Figures and Tables

**Table 1:** Hydrogen-bond geometry (Å, °)

*D*—H⋯*A*	*D*—H	H⋯*A*	*D*⋯*A*	*D*—H⋯*A*
N1—H1*A*⋯Cl1	0.89	2.30	3.122 (3)	153
N1—H1*B*⋯Cl1^i^	0.89	2.32	3.097 (3)	146
N1—H1*C*⋯Cl1^ii^	0.89	2.33	3.189 (3)	162
N2—H21⋯O1^iii^	0.84	2.22	2.963 (5)	147
N2—H22⋯O2^iv^	0.85	2.17	3.019 (5)	178

Symmetry codes: (i) 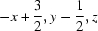; (ii) 
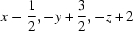
; (iii) 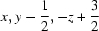; (iv) 
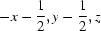
.

**Fig. 1.**
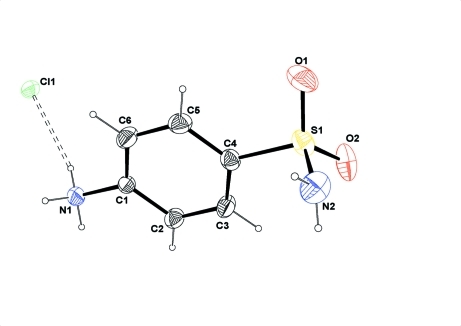
Chemical diagram of the title compound.

**Fig. 2.**
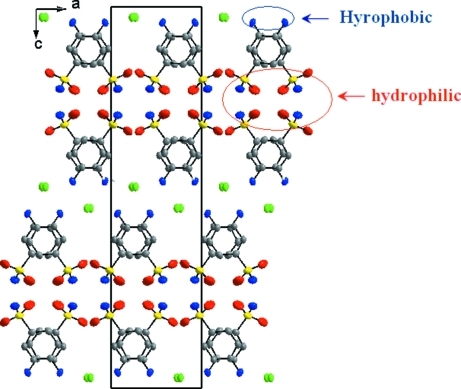
Part of the crystal structure illustrating the alternating layers of hydrophobic and hydrophilic zones, viewed along the b axis. H atoms have been omitted for clarity.

**Fig. 3.**
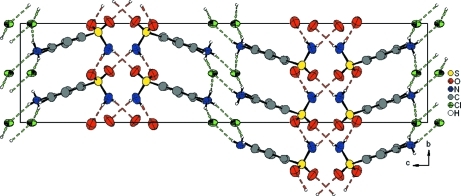
The crystal packing of the title compound, viewed along the a axis showing the N—H···Cl/O interactions (dotted line). H atoms not involved in hydrogen bonding (dashe dlines) have been omitted for clarity. Displacement ellipsoids are drawn at 50%probability level.

## supplementary crystallographic information

### Comment

The diamine compounds are important in biologically active natural products (Pasini & Zunino, 1987; Otsuka *et al.*, 1990), in medicinal chemistry (Michalson & Smuszkovicz, 1989; Reedijk, 1996). They are also used as chiral auxiliaries and chiral ligands in asymmetric catalysis (Blaser, 1992). Here, a new member of this family, (C_6_H_9_N_2_O_2_S)^+^.Cl^-^, is presented.

It was obtained during our investigations in organic chloride hybrids field. The crystal structure of the title compound contains a discrete cation with a protonated amino group (C_6_H_9_N_2_O_2_S)^+^.Cl^-^ and a halide anion (Fig 1).

The molecular structure including both terminal N atoms has an all-trans conformation. The nitrogen N1 position is protonated in this structure and participates in hydrogen bonding with the chlorine anions. The layered crystal packing of 4-sulfonamidoanilinium chloride is shown in Fig 2. The cations form alterning layers of hydrophobic and hydrophilic zones along the c axis.

The chloride ions are located in the interlayer space. Two types of classical hydrogen bonds are observed: N—H···Cl and N—H···O . These interaction bonds link the cations and the anions together, forming a three-dimensional network and reinforcing the ionic structure cohesion ((Fig 3, Table 1). The organic cations interacts with the Cl^-^ anion via hydrogen bonds, with N1—H···Cl distances ranging between 3.098 (3) Å and 3.191 (3) Å.

The packing is further consolidated through π-π stacking between symetry related benzene (3/2-x,-1/2+y,z) rings with centroid-to-centroid distance of 3.890 (2) Å and centroid-to-plane of 3.65 Å resulting in a slippage of 21°.

Selected geometrical parameters in (C_6_H_9_N_2_O_2_S)^+^ cation agree with those found in similar compounds, such as 4-aminobenzenesulfonamide (C_6_H_8_N_2_O_2_S) (Gelbrich *et al.*, 2008), 4-homosulfanilamide hydrochloride (Chatterjee *et al.*, 1981), bis(4-Aminosulfonyl)benzeneammonium hexafluorosilicate ( Gelmboldt *et al.*,2004) and 4-sulfonamidoanilinium 3,5-dinitrosalicylate (C_6_H_9_N_2_O_2_S^+^.C_7_H_3_N_2_O_7_) (Smith *et al.*, 2001).

### Experimental

Colourless crystals of 4-sulfonamidoanilinium chloride suitable for single crystal X-ray analysis were obtained by slow evaporation at room temperature of an ethanol solution of sulphanilamide (Fluka, Purity >97%) and hydrochloric acid.

### Refinement

All H atoms attached to N were located in a difference map and allowed to refine freely.

H atoms attached to C atoms were fixed geometrically and treated as riding with C—H = 0.93Å with Uiso(H) = 1.2Ueq(C).

### Crystal data

**Table:**

C_6_H_9_N_2_O_2_S^+^·Cl^−^	*F*(000) = 864
*M**_r_* = 208.66	*D*_x_ = 1.517 Mg m^−^^3^
Orthorhombic, *P**b**n**b*	Mo *K*α radiation, λ = 0.71073 Å
Hall symbol: -P 2bc 2ab	Cell parameters from 25 reflections
*a* = 7.4608 (2) Å	θ = 10–15°
*b* = 7.7278 (2) Å	µ = 0.61 mm^−^^1^
*c* = 31.694 (2) Å	*T* = 298 K
*V* = 1827.35 (13) Å^3^	Prism, colourless
*Z* = 8	0.3 × 0.2 × 0.1 mm

### Data collection

**Table:**

Enraf–Nonius CAD-4 diffractometer	1442 reflections with *I* > 2σ(*I*)
Radiation source: fine-focus sealed tube	*R*_int_ = 0.033
graphite	θ_max_ = 27.0°, θ_min_ = 2.6°
Non–profiled ω/2θ scans	*h* = −9→2
Absorption correction: ψ scan (North *et al.*, 1968)	*k* = −1→9
*T*_min_ = 0.982, *T*_max_ = 0.994	*l* = −1→40
2906 measured reflections	2 standard reflections every 120 min
1989 independent reflections	intensity decay: none

### Refinement

**Table:**

Refinement on *F*^2^	Primary atom site location: structure-invariant direct methods
Least-squares matrix: full	Secondary atom site location: difference Fourier map
*R*[*F*^2^ > 2σ(*F*^2^)] = 0.042	Hydrogen site location: inferred from neighbouring sites
*wR*(*F*^2^) = 0.182	H-atom parameters constrained
*S* = 1.07	*w* = 1/[σ^2^(*F*_o_^2^) + (0.1231*P*)^2^ + 0.2341*P*] where *P* = (*F*_o_^2^ + 2*F*_c_^2^)/3
1989 reflections	(Δ/σ)_max_ = 0.001
110 parameters	Δρ_max_ = 0.42 e Å^−^^3^
0 restraints	Δρ_min_ = −0.44 e Å^−^^3^

### Special details

**Table:**

Experimental. Number of psi-scan sets used was 5 Theta correction was applied. Averaged transmission function was used. No Fourier smoothing was applied (North *et al.*, 1968).
Geometry. All esds (except the esd in the dihedral angle between two l.s. planes) are estimated using the full covariance matrix. The cell esds are taken into account individually in the estimation of esds in distances, angles and torsion angles; correlations between esds in cell parameters are only used when they are defined by crystal symmetry. An approximate (isotropic) treatment of cell esds is used for estimating esds involving l.s. planes.
Refinement. Refinement of *F*^2^ against ALL reflections. The weighted *R*-factor wR and goodness of fit *S* are based on *F*^2^, conventional *R*-factors *R* are based on *F*, with *F* set to zero for negative *F*^2^. The threshold expression of *F*^2^ > σ(*F*^2^) is used only for calculating *R*-factors(gt) etc. and is not relevant to the choice of reflections for refinement. *R*-factors based on *F*^2^ are statistically about twice as large as those based on *F*, and *R*- factors based on ALL data will be even larger.

### Fractional atomic coordinates and isotropic or equivalent isotropic displacement parameters (Å^2^)

**Table:**

	*x*	*y*	*z*	*U*_iso_*/*U*_eq_	
S1	−0.01848 (14)	0.91801 (12)	0.80899 (3)	0.0494 (3)	
O1	0.0885 (5)	1.0213 (4)	0.78197 (10)	0.0826 (11)	
O2	−0.1835 (5)	0.9862 (4)	0.82564 (9)	0.0718 (10)	
N1	0.4306 (4)	0.7241 (3)	0.95815 (9)	0.0394 (6)	
H1A	0.4863	0.8185	0.9675	0.059*	
H1B	0.5114	0.6443	0.9512	0.059*	
H1C	0.3596	0.6828	0.9783	0.059*	
N2	−0.0703 (5)	0.7452 (5)	0.78407 (10)	0.0623 (9)	
H21	0.0127	0.7023	0.7694	0.075*	
H22	−0.1381	0.6733	0.7963	0.075*	
C1	0.3224 (4)	0.7679 (3)	0.92096 (9)	0.0317 (6)	
C2	0.1373 (4)	0.7587 (4)	0.92407 (10)	0.0402 (7)	
H2	0.0833	0.7210	0.9489	0.048*	
C3	0.0345 (4)	0.8064 (5)	0.88976 (10)	0.0433 (7)	
H3	−0.0898	0.8026	0.8915	0.052*	
C4	0.1162 (4)	0.8597 (4)	0.85296 (9)	0.0368 (7)	
C5	0.3026 (5)	0.8677 (4)	0.84957 (11)	0.0438 (8)	
H5	0.3563	0.9039	0.8246	0.053*	
C6	0.4060 (4)	0.8208 (4)	0.88399 (11)	0.0444 (7)	
H6	0.5304	0.8246	0.8824	0.053*	
Cl1	0.72981 (10)	1.00078 (9)	0.97197 (3)	0.0408 (3)	

### Atomic displacement parameters (Å^2^)

**Table:**

	*U*^11^	*U*^22^	*U*^33^	*U*^12^	*U*^13^	*U*^23^
S1	0.0590 (6)	0.0496 (5)	0.0396 (5)	0.0060 (4)	−0.0092 (4)	0.0052 (4)
O1	0.097 (3)	0.084 (2)	0.066 (2)	−0.0156 (19)	−0.015 (2)	0.0379 (16)
O2	0.0707 (18)	0.084 (2)	0.0607 (19)	0.0399 (17)	−0.0180 (16)	−0.0076 (15)
N1	0.0340 (12)	0.0321 (13)	0.0521 (16)	−0.0016 (10)	−0.0096 (11)	0.0030 (11)
N2	0.067 (2)	0.075 (2)	0.0450 (17)	−0.0057 (18)	−0.0046 (16)	−0.0158 (15)
C1	0.0284 (12)	0.0237 (12)	0.0432 (16)	0.0010 (11)	−0.0005 (12)	−0.0017 (11)
C2	0.0327 (15)	0.0508 (17)	0.0373 (15)	−0.0017 (14)	0.0024 (12)	0.0058 (13)
C3	0.0273 (13)	0.0593 (19)	0.0431 (18)	0.0005 (14)	−0.0008 (13)	0.0031 (15)
C4	0.0395 (16)	0.0311 (14)	0.0397 (17)	0.0025 (12)	−0.0044 (13)	−0.0011 (12)
C5	0.0416 (17)	0.0449 (18)	0.0450 (19)	−0.0025 (14)	0.0098 (13)	0.0050 (14)
C6	0.0306 (14)	0.0488 (18)	0.0536 (19)	−0.0019 (14)	0.0052 (14)	0.0001 (15)
Cl1	0.0310 (4)	0.0328 (5)	0.0586 (6)	−0.0038 (3)	0.0016 (3)	0.0013 (3)

### Geometric parameters (Å, °)

**Table:**

S1—O1	1.417 (3)	C1—C2	1.386 (4)
S1—O2	1.439 (4)	C1—C6	1.389 (4)
S1—N2	1.599 (3)	C2—C3	1.381 (4)
S1—C4	1.776 (3)	C2—H2	0.9300
N1—C1	1.468 (4)	C3—C4	1.379 (4)
N1—H1A	0.8900	C3—H3	0.9300
N1—H1B	0.8900	C4—C5	1.396 (5)
N1—H1C	0.8900	C5—C6	1.384 (5)
N2—H21	0.8433	C5—H5	0.9300
N2—H22	0.8457	C6—H6	0.9300
			
O1—S1—O2	119.8 (2)	C6—C1—N1	119.9 (3)
O1—S1—N2	107.9 (2)	C3—C2—C1	118.9 (3)
O2—S1—N2	106.3 (2)	C3—C2—H2	120.5
O1—S1—C4	107.35 (18)	C1—C2—H2	120.5
O2—S1—C4	106.81 (16)	C4—C3—C2	120.0 (3)
N2—S1—C4	108.23 (17)	C4—C3—H3	120.0
C1—N1—H1A	109.5	C2—C3—H3	120.0
C1—N1—H1B	109.5	C3—C4—C5	121.2 (3)
H1A—N1—H1B	109.5	C3—C4—S1	119.3 (2)
C1—N1—H1C	109.5	C5—C4—S1	119.5 (2)
H1A—N1—H1C	109.5	C6—C5—C4	118.9 (3)
H1B—N1—H1C	109.5	C6—C5—H5	120.5
S1—N2—H21	115.1	C4—C5—H5	120.5
S1—N2—H22	117.9	C5—C6—C1	119.4 (3)
H21—N2—H22	115.7	C5—C6—H6	120.3
C2—C1—C6	121.5 (3)	C1—C6—H6	120.3
C2—C1—N1	118.6 (3)		

### Hydrogen-bond geometry (Å, °)

**Table:**

*D*—H···*A*	*D*—H	H···*A*	*D*···*A*	*D*—H···*A*
N1—H1A···Cl1	0.89	2.30	3.122 (3)	153
N1—H1B···Cl1^i^	0.89	2.32	3.097 (3)	146
N1—H1C···Cl1^ii^	0.89	2.33	3.189 (3)	162
N2—H21···O1^iii^	0.84	2.22	2.963 (5)	147
N2—H22···O2^iv^	0.85	2.17	3.019 (5)	178

Symmetry codes: (i) −*x*+3/2, *y*−1/2, *z*; (ii) *x*−1/2, −*y*+3/2, −*z*+2; (iii) *x*, *y*−1/2, −*z*+3/2; (iv) −*x*−1/2, *y*−1/2, *z*.
